# Efficacy and safety analysis of combination therapy based on mitoxantrone hydrochloride liposome injection (Lipo-MIT) in relapsed/refractory NK/T-cell lymphoma

**DOI:** 10.3389/fonc.2024.1396819

**Published:** 2024-06-21

**Authors:** Xing-long Wang, He-nan Wang, Lei Yang, Jing Yang, Jia Cong, Zhi-hui Song, Liang Wang

**Affiliations:** ^1^ Department of Pharmacy, Beijing Tongren Hospital, Capital Medical University, Beijing, China; ^2^ Department of Hematology, Beijing Tongren Hospital, Capital Medical University, Beijing, China

**Keywords:** NK/T-cell lymphoma, liposomal mitoxantrone, pegaspargase, skin hyperpigmentation, immunotherapy

## Abstract

**Background:**

Currently, there is no standard treatment for relapsed/refractory NK/T-cell lymphoma (NKTCL). Liposomal mitoxantrone (Lipo-MIT) showed good anti-tumor effect in patients with NKTCL, breaking the limitation of natural resistance of NKTCL to anthracyclines. To further improve the efficacy, we tried a combination therapy based on Lipo-MIT in patients with relapsed/refractory NKTCL.

**Methods:**

12 patients with relapsed/refractory NKTCL were enrolled in this retrospective study, all of whom had previously received pegaspargase-based treatments. The salvage treatment was a combination regimen based on Lipo-MIT. The efficacy was evaluated after every two cycles.

**Results:**

11 patients had stage IV NKTCL, and all but one patients had an NRI score of ≥3. The median previous lines of treatment was two (range, 1–4), and five patients were refractory to their last line of treatment. The best response rates were as follows: complete response (CR) in five (41.7%) patients, partial response in five (41.7%) patients, stable disease in one (8.3%) patient, and progressive disease in one (8.3%) patient. At a median follow-up of four months (range, 2–14), seven patients died, with a median PFS of five months and a median OS of seven months. The six-month PFS and OS rate was 44.4% and 52.1%, respectively. All patients had suffered from side effects, among which myelosuppression was most reported. Nine patients had grade three or more myelosuppression, and the median recovery time from myelosuppression was 14 days (2–35 days). Five patients had obvious skin hyperpigmentation, and the CR rate was significantly higher compared with those without skin hyperpigmentation (80% vs. 14.3%, p=0.023). Other side effects included liver insufficiency (N=4), coagulation dysfunction (N=4), acute pancreatitis (N=2), and immunotherapy-related adverse effects (irAEs, N=2).

**Conclusion:**

Combination therapy based on Lipo-MIT has a high remission rate for relapsed/refractory NKTCL, but the duration of remission needs to be further extended. Lipo-MIT has obvious myelosuppression toxicity, and active supportive therapy should be given when combined with other cytotoxic drugs.

## Introduction

Natural killer/T-cell lymphoma (NKTCL) is an aggressive entity of non-Hodgkin lymphoma (NHL) and associated with Epstein-Barr virus (EBV) infection, which is prevalent in East Asia and South America (1). The prognosis of early stage NKTCL has been improved significantly since the use of combination of asparaginase-based chemotherapy and radiotherapy. Currently, the five-year overall survival (OS) rate for early stage NKTCL is more than 80% using various pegaspargase-based chemotherapy and radiotherapy (2). However, great challenges still exist for advanced stage and relapsed/refractory (R/R) NKTCL, especially those who failed first-line pegaspargase-based treatments. Immunotherapy targeting programmed cell death receptor 1 (PD-1)/programmed cell death ligand 1 (PD-L1) has shown promising efficacy towards R/R-NKTCL, with ORR ranging from 38%-75% (3, 4), but complete response (CR) rate and duration of response need further improvement.

Anthracyclines are commonly prescribed chemotherapy agents for lymphoma. However, NKTCL is considered to be primarily resistant to anthracyclines due to overexpression of multi-drug resistance (MDR) gene (5, 6). Mitoxantrone, a synthetic anthracycline agent, could induce DNA lesions, interfer RNA, and inhibit topoisomerase II to exerting anti-lymphoma effects. In recent years, the advent of chemotherapy drugs with liposomal formulation enhanced anti-tumor efficacy and reduced toxicity. Mitoxantrone hydrochloride liposome injection (Lipo-MIT) is approved in China for the treatment of relapsed or refractory peripheral T-cell lymphoma patients who have received at least one line standard therapy previously. Lipo-MIT showed good anti-tumor effect in patients with NKTCL subtype, with an ORR of 42.9%, which broke the limitation of natural resistance of NKTCL to anthracyclines (7). To further improve the efficacy, we tried a combination therapy based on Lipo-MIT in patients with relapsed/refractory NKTCL. Herein, we reported the efficacy and safety analysis in 12 consecutive patients with R/R-NKTCL.

## Materials and methods

### Patients

In this retrospective study, we enrolled all R/R-NKTCL patients who were treated with Lipo-MIT based treatments in Beijing Tongren Hospital from January 2022 to October 2023. All these patients were diagnosed as NKTCL according to the WHO classification (8), and failed previous pegaspargase-based chemotherapy. This study was approved by the Ethics Committee of Beijing Tongren Hospital (No. TREC2022-KY103), and written informed consent was exempted from all patients due to anonymity of all personal information.

### Treatments and assessments

Lipo-MIT based regimens were used for all patients: Lipo-MIT+pegaspargase+dexamethasone (MPD, n=3); MPD+anti-PD1 immunotherapy (MPD+PD1, n=3); MPD+etoposide (MPD+VP16, n=2); MPD+brentuximab vedotin (MPD+BV, n=1); Lipo-MIT+anti-PD1 immunotherapy (MIT+PD1, n=1); Lipo-MIT+PD1+chidamide, n=1; Lipo-MIT+PD1+BV, n=1. For patients who were refractory to pegaspargase, pegaspargase was not used. However, for those who were sensitive to pegaspargase in the first-line setting, pegaspargase was added to Lipo-MIT, aiming to get better outcomes. In all these patients, Lipo-MIT was given at the dosage of 16–20mg/m^2^ (no more than 30mg per time), which was repeated every 21 days. Pegaspargase was given at 2500IU/m^2^ (no more than 3750IU per time), repeated every 21 days. Dexamethasone was given at 20mg/day for four days. Tislelizumab (anti-PD1 immunotherapy) was given at a fixed dosage of 200mg per time, repeated every 21 days. For patients with concurrent hemophagocytic lymphohistiocytosis (HLH), etoposide was added at a dosage of 100mg/m^2^/day for three days. For those with positive expression of CD30, BV was added at a dosage of 1.8mg/kg body weight, repeated every 21 days. A maximum of six cycles of Lipo-MIT based treatment were scheduled, and consolidation with allogeneic stem cell transplantation or maintenance therapy with immunotherapy or chidamide were given at the choice of the treating physicians. Efficacy was assessed every two cycles using MRI or PET-CT scan according to revised response criteria for malignant lymphoma criteria (9), and safety profiles were documented during each cycle. Adverse events (AE) were graded according the Common Terminology Criteria for Adverse Events (CTCAE), version 5.0.

### Statistical analysis

We reported the best response and time to best response, which was defined as time from the date of treatment initiation to the date of best response documentation. Progression free survival (PFS) was defined as time from the date of treatment initiation to the date of confirmed disease progression, death, or last follow up, whichever came first. Overall survival (OS) was defined as time from the date of treatment initiation to the date of death for any reason, or last follow up, whichever came first. Correlations between skin hyperpigmentation and treatment responses were evaluated using the Chi-Square Test. Differences between the results of comparative tests were considered significant if the two-sided p-value was less than 0.05.

## Results

### Patient characteristics

The patient characteristics were shown in **Table 1**. 12 patients were enrolled in this study, and seven were male patients, with a median age of 52 years old (range, 17–70). All but one patient had stage IV disease, and nine of them suffered from repeating high fever, among whom three patients had concurrent HLH. According to the nomogram-revised risk index (NRI) (10), 11 patients were defined as high or very high risk (NRI≥3). The median previous lines of treatment was two (range, 1–4), and five patients were refractory to their last line of treatment. Six patients had detectable plasma EBV-DNA load (742–88710 copies/mL), and seven patients had more than three extranodal lesions. One patient had disease relapse less than half year after autologous stem cell transplantation, and four patients failed previous PD1 inhibitors.

**Table 1:** Clinical characteristics and treatment outcomes in patients with NK/T-cell lymphoma receiving Lipo-MIT-based therapy.

Variables	Age (years old)	Gender	ECOG score	Ann-Arbor Stage	NRI Score	Prior lines	Prior regimens and best response	Lipo-MIT-based regimens	No. of cycles	AE of MIT-based treatment	Best response	Time to best response	PFS since MIT-based Treatment	OS since MIT-based treatment	OS from diagnosis	Survival status
Pt 1	67	M	1	IVB	3	2	1.CHOPL+PD1(PR); 2.P-GEMOX+PD1(PR)	MPD	3	Grade 4 cytopenia; pancreatitis	PR	2m	3m	4m	10m	Died
Pt 2	61	M	1	IVB	3	2	1.MESA(PR) 2.GPED+PD1(CR)	MPD	6	Grade 4 cytopenia; skin hyperpigmentation	CR	3m	7m	9m	16m	Died
Pt 3	30	F	1	IVA	3	1	1.COEPL+RT(PR)	MPD+PD1	3	Grade 3 cytopenia; skin hyperpigmentation; IrAEs	CR	2m	10m+	10m+	20m+	Alive
Pt 4	53	F	1	IVB	3	1	1.P-GEMOX(CR)	MPD+PD1	4	Grade 4 cytopenia; skin hyperpigmentation	SD	2m	4m	7m	57m	Died
Pt 5	17	M	1	IVB	3	3	1.GDP-ML(PR) 2.PD1+RT(PR) 3.PD1+Chidamide(SD)	MPD+VP16	4	Grade 3 cytopenia; liver dysfunction	PR	2m	^*^14m+	^*^14m+	18m+	Alive
Pt 6	49	M	2	IVA	5	4	1.P-GEMOX(PR) 2.RT(SD) 3.BV+BeGEV(PD) 4.PD1+Decitabine+Chidamide(PD)	MPD+VP16	3	Grade 3 cytopenia; liver dysfunction	PR	2m	3m	3m	18m	Died
Pt 7	70	M	3	IVB	5	2	1.EPOCHL(CR) 2.CHOPL(SD)	MPD+PD1	2	Grade 4 cytopenia; liver dysfunction	PD	1m	2m	2m	117m	Died
Pt 8	19	M	1	IVA	4	4	1.P-GEMOX(CR) 2.PECC(PD) 3.PD1+MTX+VP16(PD) 4.PD1+Chidamide(PD)	MPD	5	Grade 2 cytopenia; pancreatitis; liver dysfunction	CR	3m	^**^5m	^**^5m	16m	Died
Pt 9	53	M	1	IB	1	1	1.P-GEMOX(PR)	Lipo-MIT+PD1	5	skin hyperpigmentation; IrAEs	CR	4m	5m+	5m+	12m+	Alive
Pt 10	51	F	3	IVB	5	1	1.GPED+PD1(PD)	Lipo-MIT+PD1+ Chidamide	2	Grade 4 cytopenia	PR	1m	2m	4m	8m	Died
Pt 11	64	F	1	IVB	4	2	1.GPED(PR) 2.MESA+ASCT(CR)	Lipo-MIT+PD1+ BV	3	Grade 4 cytopenia; skin hyperpigmentation	CR	2m	3m+	3m+	17m+	Alive
Pt 12	37	F	2	IVB	4	2	1.SMILE(CR) 2.P-GEMOX+PD1(PR)	MPD+BV	3	Grade 4 cytopenia	PR	1m	3m	3m+	36m+	Alive

*Pt 5 received allogeneic stem cell transplantation after achievement of PR and remained disease free till last follow up.

**Pt 8 died of acute severe pancreatitis while the lymphoma remained complete remission.

M, male; F, female; NRI, nomogram-revised risk index; Lipo-MIT, mitoxantrone hydrochloride liposome; MPD, MIT+pegaspargase+dexamethasone; PD1, anti-programmed cell death receptor antibody; Peg-ASP, pegaspargase; BV, brentuximab vedotin; VP16, etoposide; RT, radiotherapy; P-GEMOX, pegaspargase+gemcitabine+oxaliplatin; MESA, methotrexate+etoposide+steroid+pegaspargase; GPED, gemcitabine+pegaspargase+etoposide+dexamethasone; CHOPL, cyclophosphamide+doxorubicin+vincristine+prednisone+pegaspargase; SMILE, steroid+methotrexate+ifosphamide+asparaginase+etoposide; ASCT, autologous stem cell transplantation; CR, complete response; PR, partial response; SD, stable disease; PD, progressive disease; PFS, progression free survival; OS, overall survival; IrAEs, immunotherapy-related adverse events.

### Treatment and efficacy assessment

**Figure 1:**
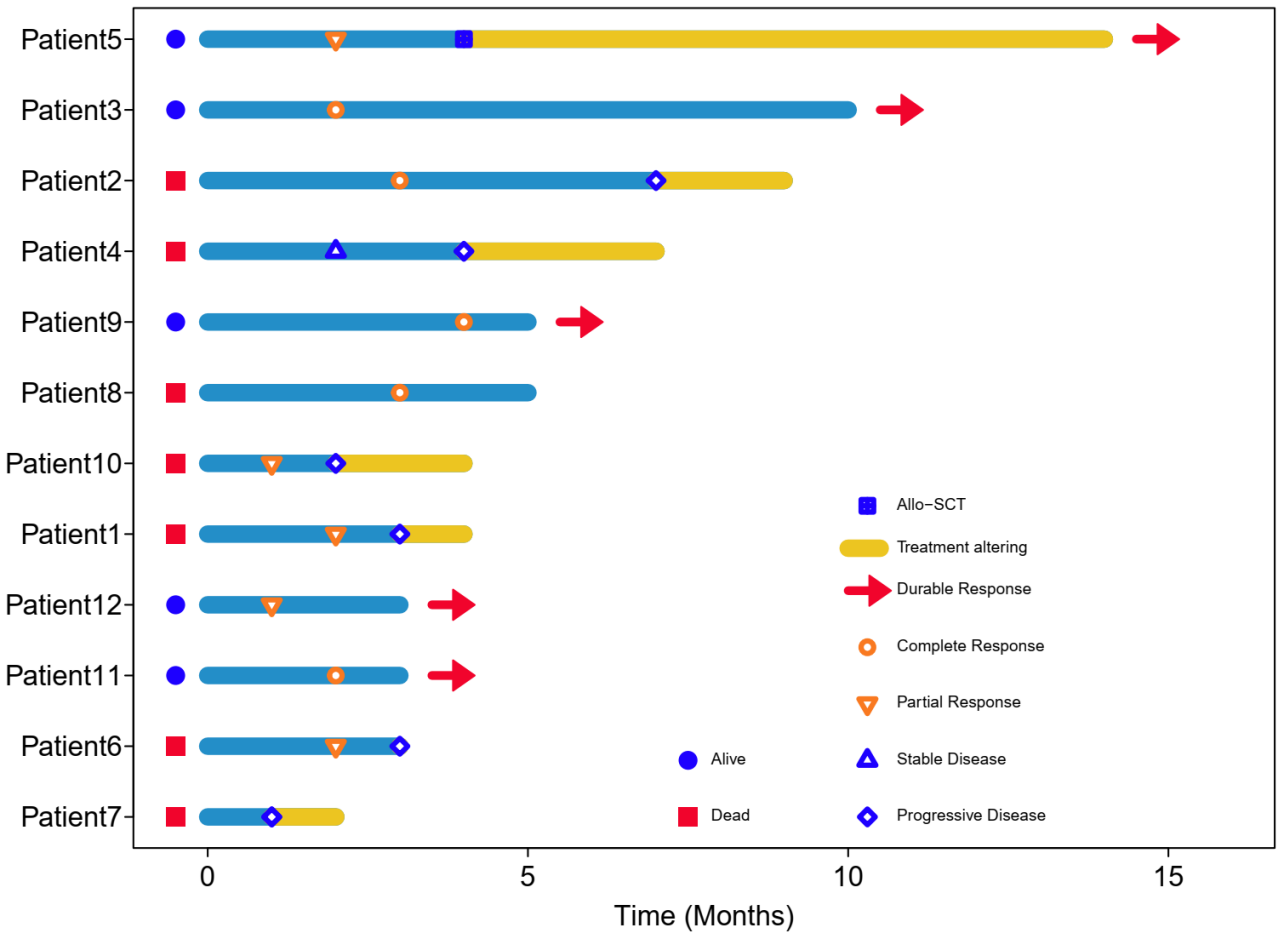
Swimming plot of patients treated with Lipo-MIT based treatments. Each bar represents one case in this study. Red head-arrow indicates that the patient still remained in disease remission and alive.

All 12 patients received Lipo-MIT based regimens, and nine patients were treated with MPD-based regimens. The median cycles for all 12 patients were three (range, 2–6), and five patients received four or more cycles of treatment. All patients were available for efficacy assessment. As is shown in **Table 1** and **Figure 1**, the best response rates were as follows: complete response (CR) in five (41.7%) patients, partial response (PR) in five (41.7%) patients, stable disease in one (8.3%) patient, and progressive disease in one (8.3%) patient. The median time to best response was two months (range, 1–4).

### Survival outcomes

**Figure 2:**
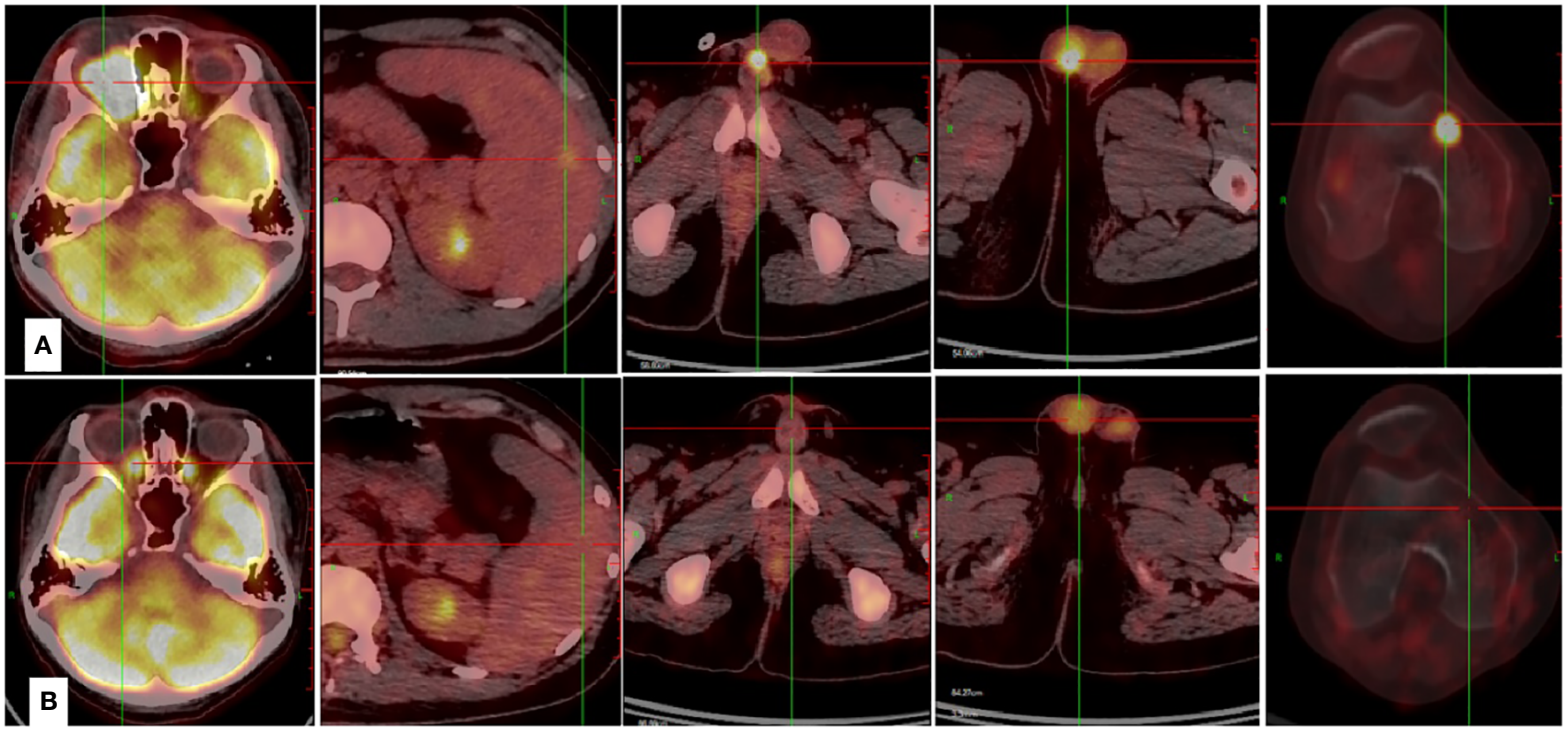
Case presentation of the efficacy achieved by Lipo-MIT based treatments. **(A)** The PET-CT scan demonstrated wide dissemination of NKTCL before the use of Lipo-MIT based treatment. **(B)** The PET-CT scan confirmed complete remission of NKTCL after four cycles of Lipo-MIT based treatment.

**Figure 3:**
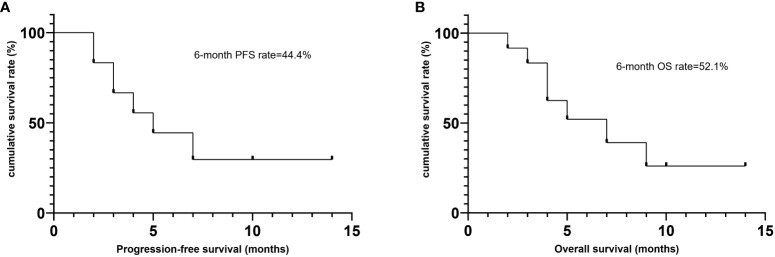
Survival outcomes of patients treated with Lipo-MIT based treamtents. **(A)** Progression free survival. **(B)** Overall survival.

Till the last follow up on October 8th 2023 and at a median follow-up of four months (range, 2–14), seven patients died, including six from disease progression and one from pegaspargase-associated acute severe pancreatitis, for whom the disease remained complete remission when died (see **Figure 2**), with a median PFS of five months and a median OS of seven months. The six-month PFS and OS rate was 44.4% and 52.1%, respectively (see **Figure 3**). One patient underwent allogeneic hematopoietic stem cell transplantation after achieving an impressive partial response and has been disease-free for more than 14 months now.

### Side effects of Lipo-MIT based treatments

All patients had suffered from side effects, among which myelosuppression (or cytopenia) was most reported. Nine patients had grade 3 or more myelosuppression, mainly manifesting as neutropenia or thrombocytopenia. The median recovery time from myelosuppression was 14 days (2–35 days). Five patients had obvious skin hyperpigmentation, especially covering the face and hand. Among the patients alive and discontinued Lipo-MIT based treatments, the side effect of skin hyperpigmentation did not disappear but alleviated. The CR rate in patients with skin hyperpigmentation was significantly higher compared with those without skin hyperpigmentation (80% vs. 14.3%, p=0.023). Other side effects included liver insufficiency (N=4), coagulation dysfunction (N=4), acute pancreatitis (N=2), and immunotherapy-related adverse effects (irAEs, N=2). One fatal adverse event was occurred in one patient. He had acute severe pancreatitis after completion of five cycles of MPD regimen, and died due to delay in treatment. None patients discontinued Lipo-MIT due to side effects except for this fatal case.

## Discussion

NKTCL is a highly aggressive EBV-associated lymphoma, and patients with advanced stage disease or who failed previous asparaginase-based treatments have dismal outcomes, especially those with extensive extranodal lesions or concurrent HLH (11). In this retrospective study, we reported the efficacy and safety profiles of Lipo-MIT based regimens for relapsed/refractory NKTCL, and demonstrated a high remission rate but relatively duration of remission. Meanwhile, Lipo-MIT has obvious myelosuppression toxicity, and active supportive therapy should be given when combined with other cytotoxic drugs.

PD1/PDL1 inhibitors have shown efficacy in relapsed/refractory NKTCL, with overall response rate (ORR) being 38%-75% and CR rate being 24%-35.9% (3, 4). Combination of sintilimab and chidamide was reported to get CR rate of 44.4% (12). No biomarkers have been identified to consistently predict responses to immunotherapy in patients with NKTCL (13). For those who failed asparaginase and immunotherapy, the survival outcomes were catastrophic. In this study, eight patients received two or more previous lines of treatment, of whom seven patients failed both asparaginase and PD1 inhibitors. Meanwhile, more than 90% of patients in our study were classified as high/very high risk groups according to the NRI scoring system. Lipo-MIT based treatment got impressive short-term efficacy in this cohort of heavily-pretreated and widely disseminated patients, with the best CR rate being 41.7% and best ORR being 83.3%. As is shown in **Figures 1** and **2**, Lipo-MIT based treatments took effect quickly, with the median time to best response being two months (range, 1–4). However, the duration of response was disappointing, with the median PFS time being only five months. Thus, consolidation strategies need to be considered after achievement of remission.

The role of hematopoietic stem cell transplantation (HSCT) has been explored in the last decade (14–16), but all were retrospective studies or single-arm prospective studies, and robust conclusions could not be reached. A French study from the Société Francophone de Greffe de Moelle et de Thérapie Cellulaire (SFGM-TC) evaluated the role of HSCT in NKTCL (14), and found that upfront HSCT did not provide benefit for patients who responded to induction treatments, but HSCT could be recommended as consolidation for relapsed patients who achieved second remission and had high risk factors. Berning et al. did a worldwide survey assessing the role of allogeneic HSCT for relapsed/refractory NKTCL (16), and reported a 3-year OS rate of 55.6%, with a potential survival plateau three years after allogeneic HSCT. In our study, one patient of 17 years old got durable remission after allogeneic HSCT, which was done when he achieved PR after Lipo-MIT based treatment. Thus, HSCT, especially allogeneic HSCT, was highly recommended for those fit patients who responded to salvage therapy, in which clinical setting Lipo-MIT based treatments could provide impressive short-term efficacy.

Previous studies have consistently found that NKTCL is primarily resistant to anthracyclines due to overexpression of p-glycoprotein on cell membrane (5). However, Lipo-MIT was shown to exert impressive anti-tumor effect in patients with NKTCL, which seemed to break the limitation of natural resistance of NKTCL to anthracyclines (7). Recently, several studies have explored the value of Lipo-MIT either as upfront induction therapy or backbone of salvage treatment, and got acceptable results (17, 18), which need to be further validated in larger cohort of patients.

Serious adverse events need to be cautious when using Lipo-MIT, especially severe myelosuppression. In our study, more than half patients had grade four cytopenia, which needed active supportive care, such as blood transfusion or hematopoietic growth factors. The median recovery time from severe myelosuppression was two weeks, and several frail patients got recovery after about one month, which obviously increased the risk of infection and bleeding. Thus, an interval of four weeks per cycle should be considered for those frail patients. Moreover, skin hyperpigmentation was common for patients who received Lipo-MIT, mainly affecting face and hands. This phenomenon could alleviate after cessation of Lipo-MIT, but did not disappear even half years later, which needs to be followed up for longer time. Skin hyperpigmentation was not specific for Lipo-MIT, and could be found in patients treated with traditional mitoxantrone, which may be caused by deposit of the drug in skin tissue. Interestingly, we found that patients with skin hyperpigmentation had significantly higher CR rates than those without it, and the underlying mechanisms need to be explored further. Overall, Lipo-MIT based treatments were well tolerated with active supportive care.

Several limitations exist for this study. Firstly, the retrospective nature could bring unavoidable confounding factors to evaluate the role of Lipo-MIT objectively. Secondly, the sample size was too small to reach robust conclusions concerning both efficacy and safety profiles. Finally, although most patients used MPD-based treatment, the regimens used in this study varied among patients, and it is difficult to infer the optimal partner drugs for Lipo-MIT.

In conclusion, this study found that combination therapy based on Lipo-MIT has a high remission rate for relapsed/refractory NKTCL, but the duration of remission needs to be further extended. Lipo-MIT has obvious myelosuppression toxicity, and active supportive therapy should be given when combined with other cytotoxic drugs. Further prospective clinical trials need to be conducted to validate and optimize the role of Lipo-MIT in the treatment of NKTCL.

## Data availability statement

The original contributions presented in the study are included in the article/supplementary material. Further inquiries can be directed to the corresponding author.

## Ethics statement

The studies involving humans were approved by Ethics Committee of Beijing Tongren Hospital. The studies were conducted in accordance with the local legislation and institutional requirements. Written informed consent for participation was not required from the participants or the participants’ legal guardians/next of kin in accordance with the national legislation and institutional requirements.

## Author contributions

XW: Validation, Writing – review & editing, Formal analysis, Methodology, Software, Data curation, Writing – original draft. HW: Validation, Data curation, Writing – review & editing, Software. LY: Writing – review & editing, Methodology, Data curation, Formal analysis. JY: Software, Methodology, Data curation, Formal analysis, Writing – review & editing. JC: Formal analysis, Methodology, Writing – review & editing. ZS: Software, Writing – review & editing, Data curation, Validation. LW: Writing – review & editing, Conceptualization, Resources, Project administration, Supervision, Funding acquisition, Software, Visualization, Data curation, Validation, Investigation, Writing – original draft, Methodology, Formal analysis.

## References

[1] Kwong YL. Natural killer-cell Malignancies: diagnosis and treatment. Leukemia. (2005) 19:2186–94. 10.1038/sj.leu.2403955 16179910

[2] Wang L Wang JW. Extranodal natural-killer T-cell lymphoma: experience from China. Lancet Haematology. (2020) 7:e441. 32470431 10.1016/S2352-3026(20)30103-4

[3] Kim SJ Lim JQ Laurensia Y Cho J Yoon SE Lee JY. Avelumab for the treatment of relapsed or refractory extranodal NK/T-cell lymphoma: an open-label phase 2 study. Blood. (2020) 136:2754–63. 10.1182/blood.2020007247 32766875

[4] Tao R Fan L Song Y Hu Y Zhang W Wang Y. Sintilimab for relapsed/refractory extranodal NK/T cell lymphoma: a multicenter, single-arm, phase 2 trial (ORIENT-4). Signal transduction targeted Ther. (2021) 6:365. 10.1038/s41392-021-00768-0 PMC8548511 34702811

[5] Wang B Li XQ Ma X Hong X Lu H Guo Y. Immunohistochemical expression and clinical significance of P-glycoprotein in previously untreated extranodal NK/T-cell lymphoma, nasal type. Am J hematology. (2008) 83:795–9. 10.1002/ajh.21256 18756548

[6] Wang L Xia ZJ Huang HQ Lu Y Zhang YJ. Cyclophosphamide, doxorubicin, vincristine, and prednisone (CHOP) in the treatment of stage IE/IIE extranodal natural killer/T cell lymphoma, nasal type: 13-year follow-up in 135 patients. Int J hematology. (2012) 96:617–23. 10.1007/s12185-012-1174-y 22983648

[7] Gao Y Huang H Wang X Bai B Huang Y Yang H. Safety and efficacy of mitoxantrone hydrochloride liposome in patients with relapsed or refractory peripheral T-cell lymphoma and extranodal NK/T-cell lymphoma: A prospective, single-arm, open-label, multi-center, phase II Clinical trial. Blood. (2020) 136:36–7.

[8] Chan JKC Quintanilla-Martinez L Ferry JA Peh SC. Extranodal NK/T-cell lymphoma, nasal type. In: Swerdlow SH Campo E Harris NL, editors. World health organization classification of tumors: pathology and genetics of tumors of haematopoietic and lymphoid tissues. IARC Press, Lyon, France (2008). p. 285–8.

[9] Cheson BD Pfistner B Juweid ME Gascoyne RD Specht L Horning SJ. Revised response criteria for Malignant lymphoma. J Clin oncology: Off J Am Soc Clin Oncol. (2007) 25:579–86. 10.1200/JCO.2006.09.2403 17242396

[10] Chen SY Yang Y Qi SN Wang Y Hu C He X. Validation of nomogram-revised risk index and comparison with other models for extranodal nasal-type NK/T-cell lymphoma in the modern chemotherapy era: indication for prognostication and clinical decision-making. Leukemia. (2021) 35:130–42. 10.1038/s41375-020-0791-3 PMC7787971 32152465

[11] Fu R Liang Y Wei L Liu X Piao Y Wang L. Combination of gemcitabine, pegaspargase, etoposide, and dexamethasone (GPED) in treatment of advanced extranodal NK/T-cell lymphoma. Chin Med J. (2023) 136:732–4. 10.1097/CM9.0000000000002570 PMC10129123 36939235

[12] Gao Y Huang H Wang X Bai B Zhang L Xiao Y. Anti-PD-1 antibody (Sintilimab) plus histone deacetylase inhibitor (Chidamide) for the treatment of refractory or relapsed extranodal natural killer/T cell lymphoma, nasal type (r/r-ENKTL): preliminary results from a prospective, multicenter, single-arm, phase ib/II trial (SCENT). Blood. (2020) 136:39–40.

[13] Wang L Wang JW. PD-1 blockade in extranodal NK/T-cell lymphoma: who is in charge? Leukemia. (2020) 34:3432–3. 10.1038/s41375-020-01046-8 33009482

[14] Philippe Walter L Couronné L Jais JP Nguyen PD Blaise D Pigneux A. Outcome after hematopoietic stem cell transplantation in patients with extranodal natural killer/T-Cell lymphoma, nasal type: A French study from the Société Francophone de Greffe de Moelle et de Thérapie Cellulaire (SFGM-TC). Am J hematology. (2021) 96:834–45. 10.1002/ajh.26200 33864708

[15] Liu C Ding H Zhu Q Liu P Zhu Y Wang L. Induction with MEDA regimen and consolidation with Auto-HSCT for stage IV NKTCL patients: A prospective multicenter study. Int J cancer. (2022) 151:752–63. 10.1002/ijc.34055 35489026

[16] Berning P Schmitz N Ngoya M Finel H Boumendil A Wang F. Allogeneic hematopoietic stem cell transplantation for NK/T-cell lymphoma: an international collaborative analysis. Leukemia. (2023) 37:1511–20. 10.1038/s41375-023-01924-x PMC10166457 37157017

[17] Cai Q Xia Y Wang L Huang H Wang J Cai J. Combination of mitoxantrone hydrochloride liposome with tislelizumab in patients with relapsed or refractory NK/T cell lymphoma: A phase ib/II Clinical trial. Blood. (2023) 142:4470.

[18] Wang X Ren J Zhu H Zhang M He P. Mitoxantrone hydrochloride liposome injection combined with carmustine, etoposide, and cytarabine as conditioning of autologous hematopoietic stem cell transplantation in NHL patients:a prospective single-arm clinical trial. Blood. (2023) 142:7062.

